# Analysis of ROR1 Protein Expression in Mice with Reconstituted Human Immune System Components

**DOI:** 10.1155/2018/2480931

**Published:** 2018-04-18

**Authors:** Carol S. Leung

**Affiliations:** ^1^Department of Hematology, University College London Cancer Institute, University College London, London, UK; ^2^Ludwig Institute for Cancer Research, Nuffield Department of Medicine, University of Oxford, Oxford, UK

## Abstract

Receptor tyrosine kinase-like orphan receptor 1 (ROR1) is an oncofetal antigen expressed on multiple tumors and has no significant expression on normal human tissues. ROR1 is highly upregulated in chronic lymphocytic leukemia (CLL) B cells. NOD-scid IL2rg^−/−^ (NSG) mice engrafted with human CD34^+^ hematopoietic progenitor cells (huNSG) achieved multilineage human immune cell reconstitution including B cells, T cells, NK cells, and DCs. Like the CLL patients, huNSG mice have abnormally high percentage of CD5-expressing B cells in the periphery. In light of this, we aim to determine whether ROR1 is expressed on huNSG B cells. Using flow cytometry analysis, we found that ROR1 was highly expressed in a proportion of bone marrow, spleen, and blood B cells, which were mostly immature B cells. Transplantation of the oncogene TCL-1-transduced CD34^+^ cells in neonatal NSG mice did not increase the frequency of ROR1-expressing B cells, but the mouse with the highest engraftment of transduced cells developed a tumor-like lump consisting of a high percentage of ROR1-expressing B cells. This study highlights the potential use of huNSG mice to study B cell malignant diseases and to evaluate immunotherapeutics targeting ROR1.

## 1. Introduction

Receptor tyrosine kinase-like orphan receptor 1 (ROR1) is an oncofetal antigen expressed in a number of malignancies. The overexpression of ROR1 in malignancy was first identified on chronic lymphocytic leukemia (CLL) B cells [1] and was subsequently found in many other hematological malignancies [2–4] and solid tumors [5]. It has been shown that ROR1 could play a crucial role in tumorigenesis [6] and cell migration [7]. As ROR1 has expression on tumor cells but not on normal human tissues except at low levels in adipose tissues, parathyroid, pancreatic islet cells, and some regions of the gastrointestinal tract [8], this makes it an attractive antigen target for cancer therapy. Indeed, a number of ROR1-specific monoclonal antibodies and chimeric antigen receptor (CAR) T cells have been developed and are under testing [9, 10]. However, a preclinical small animal model is currently lacking to evaluate ROR1-targeted immunotherapies.

Immunodeficient NOD-scid IL2rg^−/−^ (NSG) mice engrafted with human fetal liver-derived CD34^+^ hematopoietic progenitor cells (huNSG) achieved multilineage human immune cell reconstitution including B cells, T cells, natural killer (NK) cells, and dendritic cells (DCs) [11]. These so called humanized mice are a powerful tool to study human infectious diseases, hematopoiesis, and model immune system tumor interaction and can be used to evaluate novel antitumor immunotherapies [12, 13]. However, incomplete B cell development in huNSG mice has been documented [14]. Like CLL patients, huNSG mice have abnormally high frequency of B cells in the periphery, and a subset of B cells expresses CD5. In light of these, we hypothesized that huNSG mice have a high proportion of ROR1^+^ B cells and could represent a ROR1^+^ tumor model *in vivo*.

Here, we evaluated ROR1 protein expression in engrafted human immune cells in 3 different cohorts of huNSG mice. We analyzed the phenotypes and characteristics of ROR1-expressing B cells. Moreover, CD34^+^ human hematopoietic progenitor cells transduced with the oncogene T cell leukemia/lymphoma 1 (TCL-1) were transplanted in neonatal NSG mice to study the effect of this oncogene on inducing ROR1-expressing tumors in huNSG mice.

## 2. Materials and Methods

### 2.1. Generation of huNSG Mice

NOD, Cg-*Prkdc^scid^ Il2rg^tm1Wjl^*/SzJ (NSG), mice were obtained from The Jackson Laboratory and raised under specific pathogen-free conditions. Human fetal liver samples were obtained from Advanced Bioscience Resources; human CD34^+^ hematopoietic progenitor cells were isolated using the human CD34 MicroBead kit (Miltenyi Biotec). HuNSG mice were generated as previously described [11]. Animal protocols were approved by the UK Home Office, and all animal experiments were performed in accordance with institutional guidelines under protocol number 70/7295. This study was also approved by the Research Ethics Committee with REC reference number 16/EE/0043.

### 2.2. Lentiviral Vector Construction and Human CD34^+^ Cell Transduction

The coding sequence of the human TCL-1 gene was synthesized by Genscript and cloned into the lentiviral vector pCCL-GFP (Addgene) with the EF1*α* promoter. This created pCCL-EF1*α*-TCL1-GFP. Lentivirus expressing TCL-1 was then prepared by cotransfecting 293T cells with pCCL-EF1*α*-TCL1-GFP, lentiviral packaging and envelope plasmids. Lentivirus-containing supernatant was harvested after 48 hours and was used to transduce human CD34^+^ cells. Frozen CD34^+^ cells were thawed and cultured in StemPro-34 SFM complete medium (Gibco) with the cytokines SCF and IL-3, in combination with the lentiviral vectors. After 24 hours of transduction, culture media were changed and cells were cultured for another 72 hours. GFP expression was evaluated by flow cytometry before injecting these cells into NSG mice.

### 2.3. Flow Cytometry

Cells were washed in PBS and stained for 20 minutes with conjugated antibodies or isotype control obtained from BioLegend, eBioscience, or BD. The following purified mouse anti-human antibodies were used for staining: anti-CD45-Pacific Blue, anti-CD45-BV605, anti-CD19-PE-Cy7, anti-CD19-APC-Cy7, CD10-BV605, anti-CD27-PE-Cy7, anti-IgD-PE, anti-ROR1-APC (clone 2A2), anti-CD5-PerCP-Cy5.5, anti-CD38-BV711, anti-CD23-FITC, anti-NKp46-FITC, anti-CD3-Pacific Blue, anti-CD4-APC-Cy7, and anti-CD8-PE. Live and dead cell staining was performed with aqua fluorescent reactive dye from Invitrogen. Peripheral blood mononuclear cells (PBMCs) from heathy donors and CLL patients were kindly provided by Dr. Vania Coelho, University College London. Flow cytometry analysis was done using a BD LSRII Fortessa using FACSDiva software (BD Biosciences), and data were analyzed with FlowJo (Tree Star).

### 2.4. B Cell Proliferation Assay

Frozen splenocytes from huNSG mice in the same reconstitution cohort were thawed and stained with 5 *μ*M CellTrace Violet (Invitrogen) and washed, and 200,000 live splenocytes were plated in 200 *μ*l of culture medium in each well of 96-well flat-bottom plates. The cells were stimulated with 5 *μ*g/ml CpG ODN 2006 (InvivoGen), 167 ng/ml pokeweed mitogen (PWM) extract (Sigma), and 1/2400 fixed *Staphylococcus aureus* cells (SAC) (Calbiochem) for 96 hours and analyzed by flow cytometry.

### 2.5. Western Blot

Untransduced or transduced CD34^+^ hematopoietic progenitor cells by lentivirus expressing TCL-1 were lysed by RIPA buffer containing protease inhibitor (Sigma). Protein extracts were separated by Bis-Tris gels and transferred to the PVDF membrane by Western blotting and probed with TCL-1-specific monoclonal antibody clone 1-21 (Cell Signaling). Goat anti-mouse IgG coupled with HRP was used as a secondary antibody. Blots were developed using the ECL kit (GE Healthcare), and protein bands were detected on X-ray film.

## 3. Results

### 3.1. ROR1 Expression on B Cells in huNSG Mice

**Figure 1:**
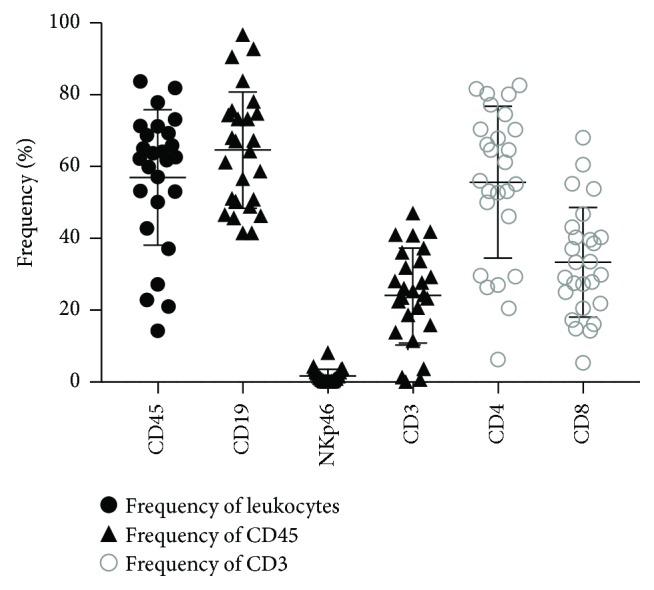
NOD-scid IL2rg^−/−^ (NSG) mice injected with fetal liver-derived CD34^+^ hematopoietic progenitor cells were reconstituted with human immune cells. Peripheral blood of reconstituted NSG mice was analyzed 3 months after injection of human hematopoietic progenitor cells. The frequencies of different immune cell compartments are indicated. Frequencies of human CD45^+^ cells within the leukocyte gate, frequencies of CD19^+^ B cells, NKp46^+^ NK cells, and CD3^+^ T cells within human CD45^+^ cells, and frequencies of CD4^+^ and CD8^+^ T cells within CD3^+^ cells are shown. Horizontal lines represent the mean and SD. Data are from 3 different reconstitution cohorts with CD34^+^ cells derived from 3 different fetal liver tissues.

**Figure 2:**
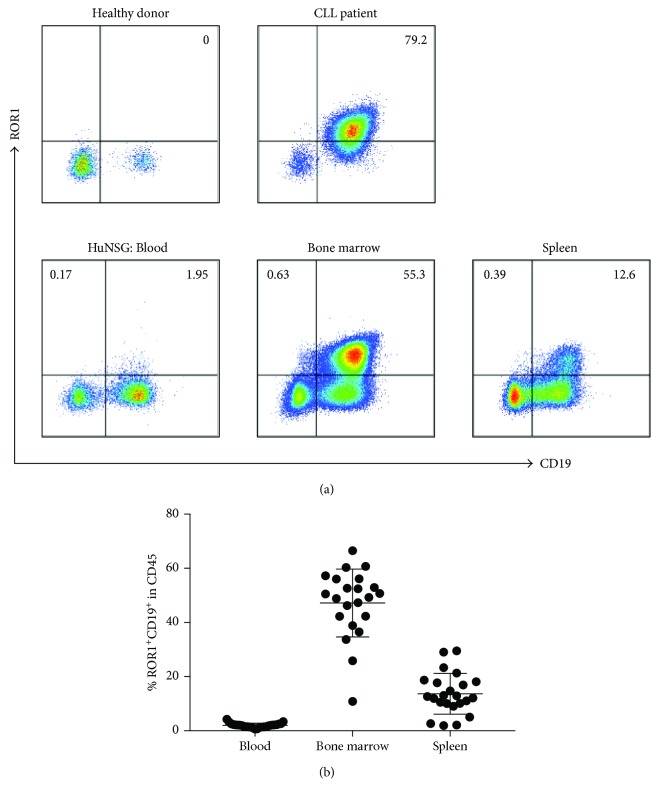
ROR1 expression on B cells in huNSG mice. (a) Flow cytometry staining of CD19^+^ and ROR1^+^ cells in PBMCs isolated from a healthy donor and a CLL patient (upper panel) and cells isolated from the blood, bone marrow, and spleen (lower panel) of huNSG mice. Samples were pregated as live cells, singlet cells positive for human CD45. The numbers indicate the frequency of CD19^+^ and ROR1^+^ cells within human CD45^+^ cells. (b) Composite data from 3 independent experiments are shown. Each data point represents one individually analyzed mouse. Horizontal lines represent the mean and SD.

We first examined the ROR1 surface expression on reconstituted human immune cells in huNSG mice. These mice were generated by engrafting newborn immunodeficient NSG mice with human fetal liver-derived CD34^+^ hematopoietic progenitor cells [11, 15]. We generated 3 cohorts of huNSG mice with human CD34^+^ hematopoietic progenitor cells derived from 3 different fetal liver tissues. Most of the huNSG mice achieved a frequency of more than 50% of human CD45^+^ cells in total leukocytes after 3 months of reconstitution, with engraftment of CD19^+^ B cells, CD3^+^ T cells, and NKp46^+^ NK cells (Figure 1). Afterwards, we investigated the ROR1 surface expression on engrafted human immune cells in huNSG mice, comparing such expression with that in a human healthy donor and a CLL patient. PBMCs from the healthy donor did not express ROR1 while a high proportion of ROR1-expressing B cells was observed in the PBMCs of the CLL patient (Figure 2(a)). Interestingly, we found a high percentage of CD19^+^ROR1^+^ B cells in huNSG mice, especially in the bone marrow and spleen. This was observed in mice from all 3 cohorts, with a mean of 47.2% in the bone marrow, 13.7% in the spleen, and 2.0% in the blood (Figure 2(b)). On the other hand, only a negligible amount of CD45^+^CD19^−^ immune cells expressed ROR1.

### 3.2. Frequency of ROR1-Expressing B Cells Maintained in huNSG Mice

**Figure 3:**
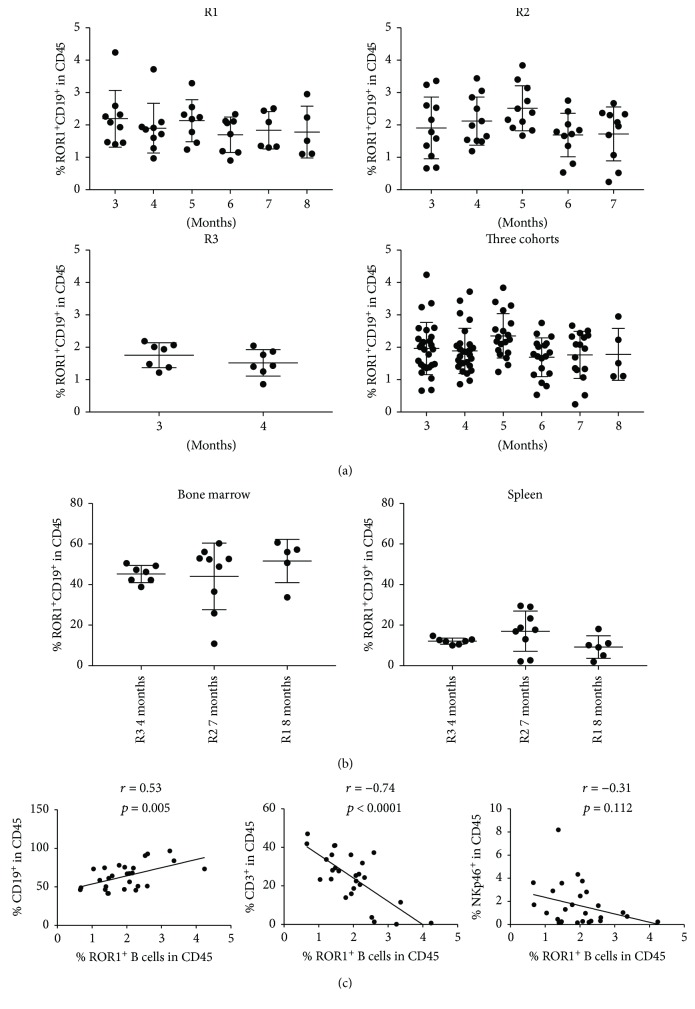
Frequency of ROR1-expressing B cells maintained in huNSG mice. (a) The frequency of ROR1^+^ B cells within human CD45^+^ cells in the blood of huNSG mice was analyzed in different time points after reconstitution. Data from 3 different cohorts of huNSG mice are shown individually (R1, R2, and R3) and as composite data (three cohorts). (b) Frequency of ROR1^+^ B cells within human CD45^+^ cells in the bone marrow (left) and spleen (right) of huNSG mice at different time points. Horizontal lines represent mean and SD. (c) Correlation of the frequencies of ROR1^+^CD19^+^ cells with the frequencies of human CD19^+^ B cells, CD3^+^ T cells, and NKp46^+^ NK cells in huNSG mice. The Pearson coefficient *r* is shown, a two-tailed statistical analysis was performed, and the *p* value is shown.

The abnormally high percentage of ROR1^+^ B cells may have been caused by the incomplete B cell development in huNSG mice [14]. A previous study has suggested that human B cell maturity improves with time following the reconstitution [16], so we tested if the frequency of ROR1^+^ B cells changes over time after the transplantation of human cells. In Figure 3(a), the percentage of ROR1^+^CD19^+^ B cells in the periphery remained largely unchanged between 3 and 8 months after human progenitor cell engraftment. This was observed in both individual cohorts and the pooled cohort. In addition, the frequency of this subset was also stable over time in the bone marrow and spleen of huNSG mice, although it should be noted that each cohort only contributed to one single time point (Figure 3(b)). Also, the reconstituted frequency of ROR1^+^CD19^+^ B cells correlated positively with CD19 reconstitution (*r* = 0.53) and negatively with CD3 reconstitution (*r* = −0.74), but to a lesser extent with NKp46^+^ NK cell reconstitution (*r* = −0.31).

### 3.3. ROR1-Expressing B Cells Were Mostly Immature B Cells

**Figure 4:**
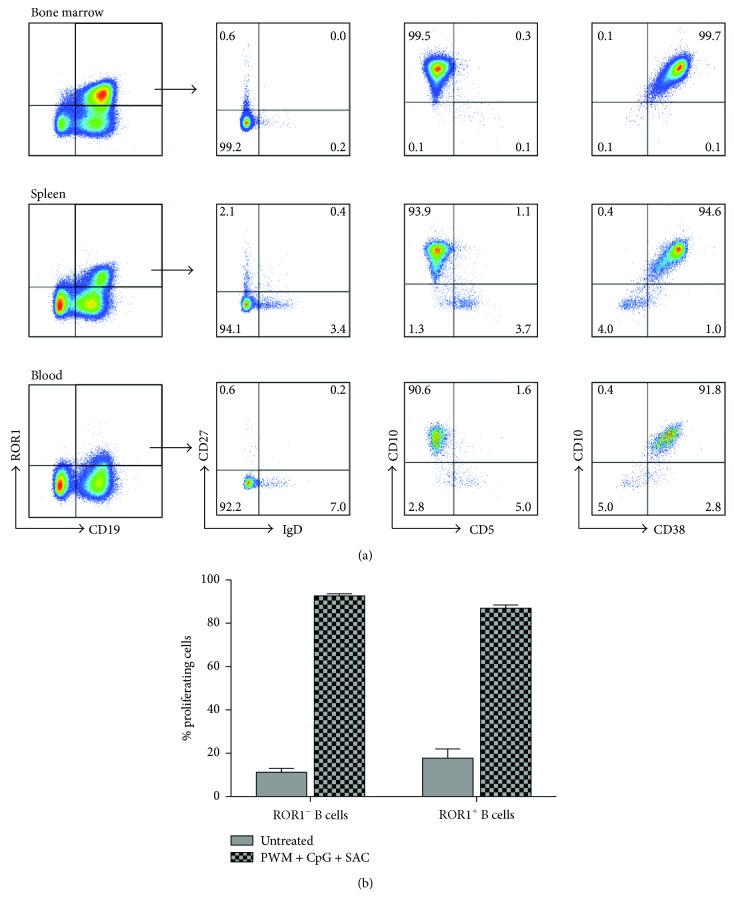
ROR1-expressing B cells were mostly immature B cells. (a) Flow cytometry staining of cells from the bone marrow, spleen, and blood of huNSG mice. Cells from the left panel were pregated as live cells, singlet cells positive for human CD45. ROR1^+^CD19^+^ cells were further analyzed for their expression of CD27, IgD, CD10, CD5, and CD38. Representative data from 3 different cohorts of huNSG mice are shown. (b) Splenocytes from huNSG mice were stained with CellTrace Violet and stimulated with PWM, CpG, and SAC or left unstimulated. After 4 days, cells were harvested and stained with CD19 and ROR1 antibodies, and percentage of proliferation of live gated CD19 B cells was measured by dye dilution by flow cytometry. The % of proliferating ROR1^+^ and ROR1^−^ B cells is shown. The graph depicts the results from 2 experiments.

We examined the phenotype of the ROR1^+^CD19^+^ B cells in huNSG mice. In the bone marrow, ROR1-expressing B cells were mainly CD27 and IgD double negative (99.2%), CD10^+^, and CD38^+^ but were CD5^−^ (Figure 4(a)). The majority of the splenic ROR1-expressing B cells were also CD27^−^IgD^−^ and CD10^+^CD38^+^, with slightly larger CD27^−^IgD^+^, CD10^−^CD5^+^, and CD10^−^CD38^−^ populations than those of the bone marrow. The peripheral blood ROR1-expressing B cells had the relatively highest frequency of CD27^−^IgD^+^, CD10^−^CD5^+^, and CD10^−^CD38^−^ population compared to those of the spleen and bone marrow, but again the majority were mainly CD27^−^IgD^−^ and CD10^+^CD38^+^cells. Although we observed a high percentage of CD5^+^ B cells in huNSG mice, these cells rarely expressed ROR1 (data not shown). These results indicated that ROR1-expressing B cells in huNSG mice were mostly immature human B cells. We next tested if the ROR1^+^ B cells can proliferate better than the ROR1^−^ B cells. It has been shown that the combination of potent B cell stimulators, PWM, CpG, and SAC, could lead to B cell activation and proliferation [17, 18]. We then stimulated the splenocytes of the huNSG mice with PWM, CpG, and SAC for 4 days and measured the proliferation by fluorescence dye dilution. The unstimulated ROR1^+^CD19^+^ B cells had a slightly higher percentage of proliferating cells than the unstimulated ROR1^−^CD19^+^ B cells (18.3% versus 11.7%), while the stimulated cells had a comparable high proliferation (over 85%).

### 3.4. Engraftment of TCL-1-Transduced Human CD34^+^ Cells in NSG Mice Could Induce ROR1-Expressing Tumors

**Figure 5:**
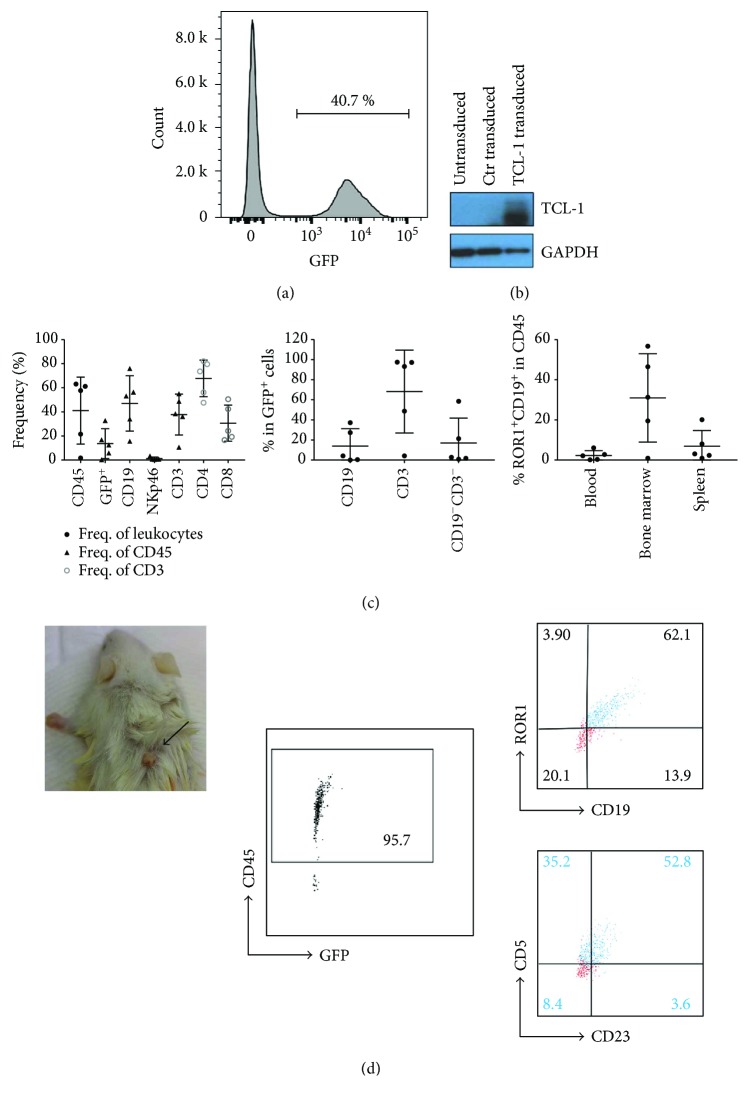
Engraftment of TCL-1-transduced human CD34^+^ cells in NSG mice could induce ROR1-expressing tumors. (a) Human CD34^+^ cells isolated from fetal liver tissues were transduced with lentivirus expressing TCL-1 GFP. Frequency of GFP-positive CD34^+^ cells after 4 days of lentiviral transduction is shown. (b) CD34^+^ cells transduced with lentivirus expressing TCL-1 (TCL-1 transduced) and control lentivirus (Ctr transduced) or untransduced were harvested and lysed 4 days after infection. Protein lysates were separated by gel electrophoresis, transferred to PVDF membranes by Western blotting, and probed with TCL-1-specific antibody. The blot was also probed for GAPDH as a loading control. (c) Peripheral blood from NSG mice transplanted with lentiviral-transduced CD34^+^ cells was analyzed 3 months after reconstitution. The frequencies of different immune cell compartments are indicated. Frequencies of human CD45^+^ cells within the leukocyte gate, frequencies of GFP^+^ cells, CD19^+^ B cells, NKp46^+^ NK cells, and CD3^+^ T cells within human CD45^+^ cells, and frequencies of CD4^+^ and CD8^+^ T cells within CD3^+^ cells are shown (left). The composition of the GFP^+^ cells is shown (middle), and the frequency of CD19^+^ and ROR1^+^ cells within human CD45^+^ cells in the blood, bone marrow, and spleen of the mice is presented (right). Horizontal lines represent mean and SD. Data are from 2 different reconstitution cohorts with CD34^+^ cells derived from the same donor. (d) The mouse with the highest GFP^+^ cells in the blood developed a tumor-like lump at the back as pointed by the arrow (left). Cells isolated from the tumor-like lump were analyzed by flow cytometry for the expression of human CD45 and GFP (left). The gated human CD45^+^ cells were further analyzed for CD19, ROR1, CD5, and CD23 expression. The ROR1^+^CD19^+^ cell population is represented in blue.

The first transgenic mouse model of CLL was generated by overexpressing the TCL-1 gene under the control of the immunoglobulin heavy chain variable region promoter and immunoglobulin heavy chain enhancer [19]. We therefore introduced the human TCL-1 gene to human CD34^+^ hematopoietic progenitor cells by lentiviral transduction. A viral 2A sequence was used for the simultaneous overexpression of both the TCL-1 gene and enhanced green fluorescent protein (GFP), so the TCL-1 transduction efficiency could be monitored by GFP expression. We achieved a transduction efficiency of around 40% (Figure 5(a)), and the expression of TCL-1 in the CD34^+^ cells was confirmed by Western blotting (Figure 5(b)). The transduced cells were used to engraft newborn NSG mice. After 3 months of engraftment, most of the mice had achieved a comparable frequency of human CD45^+^ cells in total leukocytes, with engraftment of CD19^+^ B cells, CD3^+^ T cells, and NKp46^+^ NK cells. Four out of the five reconstituted mice had GFP-positive cells in the periphery, with the majority being CD3^+^ T cells (Figure 5(c)). Two mice had a relatively higher proportion of GFP-positive B cells. However, the frequency of ROR1^+^ B cells in these mice was similar to that in the huNSG mice reconstituted using untransduced CD34^+^ cells (Figures 2(b) and 5(c)). Interestingly, the mouse with the highest engrafted GFP^+^ cells in the periphery developed a tumor-like lump after 6 months of reconstitution. This has never been observed in other cohorts of huNSG mice engrafted with unmanipulated CD34^+^ cells. Single suspension cells isolated from this lump had more than 90% CD45^+^ human cells, without GFP expression. A high proportion of the CD45^+^ cells was ROR1-expressing CD19^+^ B cells, and more than 50% of the ROR1^+^CD19^+^ cells coexpressed CD5 and CD23 (Figure 5(d)).

## 4. Discussion

In line with other findings [15, 20], our huNSG mice were reconstituted with all major subsets of immune cells after neonatal hematopoietic progenitor cell transplantation. huNSG mice have a high frequency of CD19^+^ B cells in the periphery, and over 50% of these B cells express the CD5 antigen [21] (data not shown). CLL patients also have a high frequency of CD5^+^ B cells in the periphery [22], and most of the CLL cases are positive for ROR1 surface expression [23]. Our data show that a high percentage of B cells in huNSG mice expresses ROR1, especially in the bone marrow and spleen.

HuNSG mice can also be generated using CD34^+^ hematopoietic progenitor cells isolated from umbilical cord blood or from GM-CSF-mobilized peripheral blood cells [24, 25], other than the human fetal liver. A number of studies have documented incomplete B cell development in different humanized mouse models, including the BLT mice [21, 26, 27]. If the ROR1^+^ immature B cells are indeed the by-product of incomplete B cell development in huNSG mice, it is likely that these cells are also present in other humanized mouse models. In contrast, with recent advances in using new mouse strain to generate better humanized mice with improved human B cell compartment [28, 29], we would expect to see less ROR1^+^ immature B cells in these new models.

It has been reported that human T cells were required for B cell maturation in a humanized mouse model [16]. This might explain the significant inverse correlation of ROR1^+^ B cells with T cells in our model. A higher number of reconstituted human T cells could aid B cell maturity and lead to a decrease in immature B cells. Since ROR1-expressing B cells were mostly immature B cells, the numbers decreased with increasing T cells. In the case of NK cells, we did not observe a significant inverse correlation with ROR1^+^ B cells, suggesting that NK cells did not play a role in B cell maturation in huNSG mice.

ROR1 is a novel target for cancer immunotherapy as it is overexpressed in a number of malignancies without significant expression in normal adult tissues [30]. Different approaches including CAR T cells [10], monoclonal antibodies [9, 31], and bi-specific T cell engagers (BiTEs) [32] targeting ROR1 have been developed to treat tumors. All these immunotherapies require the help of other immune cells to function. ROR1-specific CAR has to be engineered in T cells to recognize and kill ROR1^+^ target cells. Also, endogenous T cells have to be activated by BiTEs to act on ROR1^+^ tumor cells. Additionally, ROR1-specific monoclonal antibody therapy may require NK cells to mediate cytotoxicity. Therefore, an *in vivo* model consisting of human ROR1^+^ target cells and autologous immune cells would be a valuable tool to evaluate these therapies. Our data indicate that huNSG mice have a stable proportion of ROR1^+^ B cells in the peripheral blood, spleen, and bone marrow, together with functional autologous T cells and NK cells. It has been shown that huNSG T cells can be transduced and adoptively transferred back to the same host to control viral infection [33]. With this, ROR1-specific CAR T cells could be generated from huNSG mice and the efficacy can be evaluated by the removal of ROR1^+^ cells in the host. As we can deplete different subsets of immune cells in huNSG mice [15, 34], this model enables a better study of the immunotherapies.

ROR1 surface expression in B cells is detected in more than 95% of CLL cases [1]. Though we found a high percentage of huNSG B cells expressing this oncofetal antigen, these were mainly immature B cells and had a very different phenotype compared to CLL B cells. It has been reported that surface ROR1 is present at an early stage of normal B cell differentiation in human bone marrow [10]. While ROR1-expressing CLL B cells are CD5^+^CD23^+^ mature cells [35], ROR1^+^ B cells in huNSG mice are mainly CD5^−^CD23^−^ immature nonneoplastic B cells. That limits the potential use of this model to study antitumor efficacy. Moreover, this model should not be used for safety study of ROR1-directed immunotherapies because huNSG mice have a much higher frequency of ROR1^+^ immature B cells than humans.

We attempted to generate an *in vivo* CLL model by manipulating the CD34^+^ human hematopoietic progenitor cells. As the first transgenic mouse model of CLL was generated by overexpressing the human TCL-1 gene under the control of the immunoglobulin heavy chain variable region promoter and immunoglobulin heavy chain enhancer [19], we transduced CD34^+^ human hematopoietic progenitor cells with TCL-1-expressing lentivirus before injecting these cells into the neonatal mice. The reconstituted mice did not develop a CLL-like disease or other leukemic diseases. This may be due to the following reasons. First, the reconstituted GFP-positive cells, representing the engraftment of TCL-1-transduced CD34^+^ cells, were at a relatively low level, with the highest only at 32% of CD45^+^ cells in one of the five mice. Second, the TCL-1 transgenic mouse model of CLL has a delayed disease development, in which CLL-like disease is usually developed at 8–12 months of age [36], whereas our mice were only at 6-7 months of age. Third, the TCL-1 gene was expressed under the EF1*α* promoter in our model. Future study should examine if TCL-1 overexpression under the control of the immunoglobulin heavy chain variable region promoter and immunoglobulin heavy chain enhancer could lead to a CLL-like phenotype.

Although the reconstituted mice did not develop CLL-like disease, the mouse with the highest reconstituted GFP-positive cells in the blood developed a tumor-like lump. Cells isolated from the lump were human CD45^+^, and the majority were ROR1-expressing B cells. More than half of these cells coexpressed CD5 and CD23, hinting that they might be neoplastic B cells. However, these cells did not express GFP, suggesting that they might not be derived from the TCL-1-transduced CD34^+^ cells, and how TCL-1 affected the interaction of the immune cells in huNSG mice and led to the development of this tumor-like lump remains to be determined.

In order to generate a consistent ROR1^+^ tumor model in huNSG mice, we have to improve the transduction efficiency of CD34^+^ cells and achieve a higher engraftment of TCL-1-transduced CD34^+^ cells [37]. The oncogenic nature of TCL-1 is well documented [38], but the addition of ROR1 overexpression in the CD34^+^ cells should be able to promote and drive tumorigenesis in huNSG mice [39]. Moreover, it has been shown that introducing genetic changes to CD34^+^ human hematopoietic progenitor cells before injecting these cells into immunodeficient mice could generate a humanized mouse leukemic model that has recapitulating features of primary leukemia [40]. Introducing the genetic deletion of the chromosomal region 13q14, a common cytogenetic abnormality in CLL [41], could be considered.

In summary, we have shown that ROR1 protein was highly expressed in a proportion of B cells in huNSG mice; the majority of these cells were immature B cells. Transplantation of TCL-1-transduced CD34^+^ human hematopoietic progenitor cells in neonatal NSG mice did not increase the frequency of ROR1-expressing B cells, but the mouse with the highest engraftment of transduced cells developed a tumor-like lump consisting of a high percentage of ROR1-expressing B cells. Further work would be required to induce frequent and consistent ROR1^+^ tumors in huNSG mice for studying immunotherapies targeting ROR1.
